# Importance of Touch: Managing Testicular Torsion in a 28-Year-Old With Duchenne Muscular Dystrophy

**DOI:** 10.7759/cureus.22775

**Published:** 2022-03-02

**Authors:** Chaitya Desai, Kshitij Raghuvanshi, Harrypal Panesar, Vinodh Murali

**Affiliations:** 1 Urology, University Hospitals of North Midlands NHS Trust, Stoke-on-Trent, GBR; 2 Vascular Surgery, University Hospitals of North Midlands NHS Trust, Stoke-on-Trent, GBR

**Keywords:** urology, local anaesthesia, duchenne muscular dystrophy, scrotal exploration, testicular torsion

## Abstract

This case report highlights a case of testicular torsion in a man over the age of 25 with Duchenne muscular dystrophy (DMD), who presented with an atypical pain history, and a Testicular Workup for Ischaemia and Suspected Torsion (TWIST) score negative for exploration. However, based purely on the examination findings, scrotal exploration was performed and a torted testis was found. The report demonstrates that in this cohort of patients, a higher index of suspicion is needed to ensure early recognition of the condition. Furthermore, scrotal exploration can be safely conducted under local anaesthesia given the multiple cardiovascular and spinal co-morbidities attributed to DMD.

## Introduction

Duchenne muscular dystrophy (DMD) is an X-linked recessive genetic disorder which predominantly affects boys and causes severe, progressively worsening muscle weakness [1]. The disorder is the most common form of muscular dystrophy to affect children and is a result of DMD gene mutations, thus preventing the production of the muscle isoform of dystrophin (Dp427m) [1]. Patients with DMD may initially present with recurrent falls due to gait abnormalities at around two years of age [2]. By the age of 20, most patients will be wheelchair-bound [2]. A recent meta-analysis found that the median life expectancy for DMD patients was 22.0 years (95% confidence interval [CI] 21.2, 22.4) [2, 3].

Testicular torsion is a urological emergency caused by twisting of the testicle on the spermatic cord leading to time-sensitive ischaemia of testicular tissue, with an approximate 6-hour window from onset of pain to irreversible testicular damage [4]. The highest prevalence for testicular torsion is between 12 and 18 years of age, and statistics show the annual incidence rate is approximately 1 in 4000 persons under 25 years [5]. Upon assessing a patient with acute scrotal pain, if the history and physical examination findings are consistent with testicular torsion, then an emergency scrotal exploration operation must be performed.

## Case presentation

A 28-year-old man with a background of DMD, was referred to the urology team from the emergency department at 3 am for right scrotal pain. It was severe and sudden in onset, starting at around 8 pm the previous day. Initially, the patient thought the pain was in his abdomen and due to moving from his wheelchair to his bed. There were no urinary symptoms or history of trauma. The patient was apyrexial and haemodynamically stable with no nausea or vomiting. Blood tests showed a slightly raised white cell count of 13.1, but C-reactive protein was <4. On examination, there was mild tenderness in the right testis and the patient reported his pain had eased with paracetamol. The right testis was horizontal in lie, slightly harder and higher riding compared to the left. Both testes were not swollen with bilateral absent cremasteric reflexes due to the patient’s advanced DMD. Based on this he had a Testicular Workup for Ischaemia and Suspected Torsion (TWIST) score of 3 which would constitute an ultrasound scan to validate the findings. However, given the lack of specialised urological ultrasound radiographers at night-time and the highly suspicious physical examination findings, despite a low TWIST score, the decision for scrotal exploration was taken with the patient’s consent.

**Figure 1:**
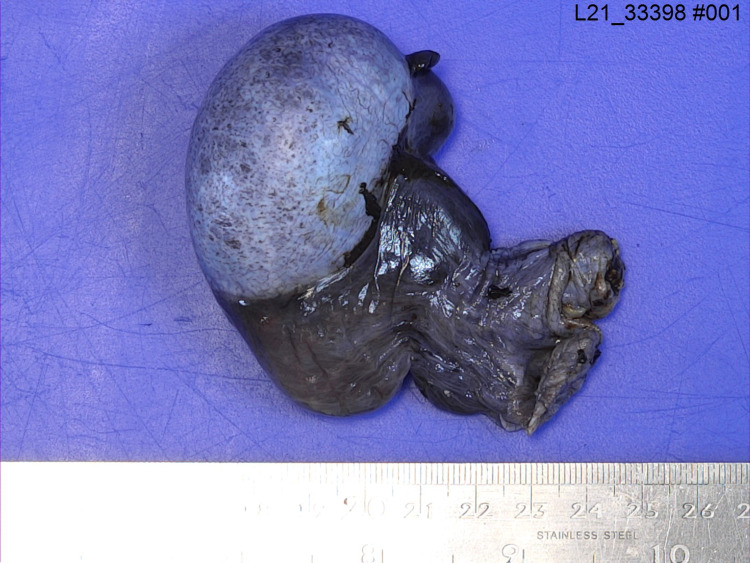
Gross pathology – Outer surface of right torted testis (medial view).

**Figure 2:**
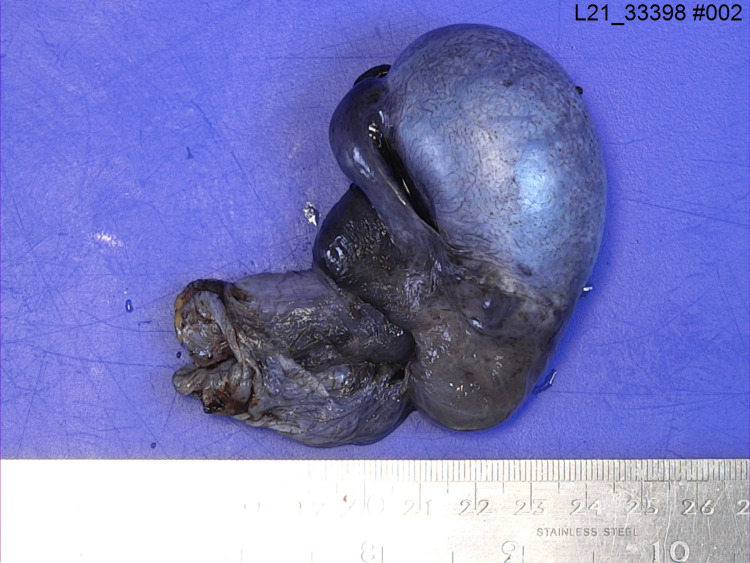
Gross pathology – Outer surface of right torted testis (lateral view).

**Figure 3:**
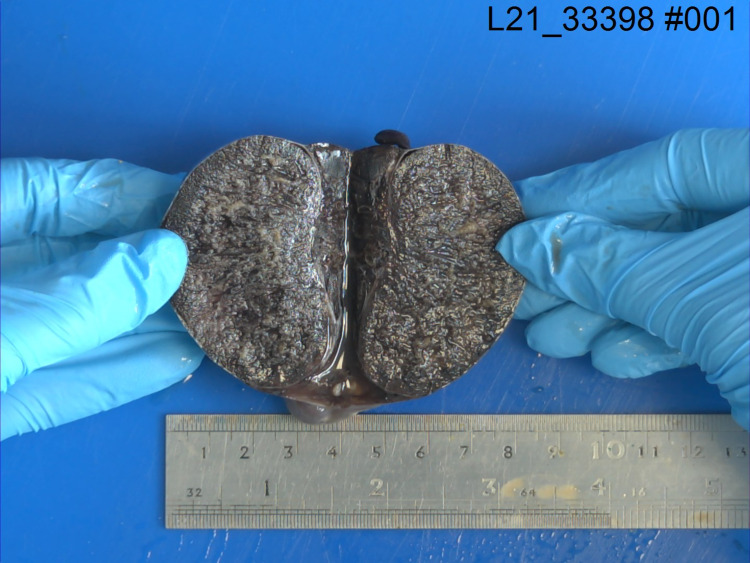
Gross pathology – Cross-sectional surface of right torted testis.

Then, following discussion with the anaesthesiology team, the patient was not unfit to withstand general anaesthesia. As a result of his DMD, the patient suffered from dilated cardiomyopathy and severe mitral regurgitation, resulting in a cardiac resynchronisation therapy implantable cardioverter-defibrillator (CRT-ICD) in-situ. The patient also used a continuous positive airway pressure (CPAP) machine at night for ventilation. Furthermore, the patient had severe scoliosis which made spinal anaesthesia unsuitable. Thus, the decision was made to use local anaesthesia only. Ultrasound guidance was used to give a bilateral spermatic cord block. On exploration, the right testis was found to be in 540 degrees torsion with oedema and black in colour (see Figures 1-3). Untwisting was done and warm saline-soaked gauze was applied for 10 minutes, but there was no change in colour. A needle was used to prick the testis in several places but there was no bleeding. The findings were discussed with the patient as he was conscious and a right orchidectomy was performed. The left testis was healthy, and a 3-point fixation was performed. There were no post-operative complications.

## Discussion

Testicular torsion was first reported in 1840, and subsequently the first review article was published by Scudder in 1901 [6]. Testicular torsion is defined as a mechanical twisting of the spermatic cord or testis due to abnormal fixation within the tunica vaginalis - resulting in testicular infarction and potential infertility [7, 8]. The two types of testicular torsion are: intravaginal and extravaginal.

Intravaginal testicular torsion is due to the rotation of the testicle on the axis of the spermatic cord within the tunica vaginalis [9]. This form of the condition commonly occurs in older children (due to periods of testicular growth during puberty) and adults. The "bell-clapper deformity" is a form of intravaginal torsion, and is a result of two main defects: (1) a pathologically high attachment of the tunica vaginalis over the spermatic cord, and (2) a breakdown of the natural posterior attachment of the testicle to the inner scrotum [10]. On the other hand, in extravaginal torsion, the twisting occurs outside the tunica vaginalis. This extravaginal form is more common in the perinatal period as the attachments of the tunica vaginalis to the scrotal wall are not fully developed. As a child develops, these attachments fortify, decreasing the chance of extravaginal torsion [11, 12].

To the best of our knowledge, we are reporting for the first time in history, the management of intravaginal torsion in a 28-year-old patient with DMD and the operation was performed under local anaesthesia. In spite of the consistent age distribution for presentation, an 82-year-old man has been reported as having testicular torsion in the literature [13]. Therefore, although over 88% of patients with testicular torsion present before the age of 25, the condition should not be excluded purely on the basis of age [14, 15]. This is the principle we followed with the patient in this report. Furthermore, even in the absence of common presenting complaints, the examination findings raised enough of a suspicion that scrotal exploration was determined as the next appropriate step of action.

It has been hypothesised that with increasing age, the decrease in testicular volume causes a relative increase in the intravaginal space, which could result in further mobility and thus torsion [16]. This phenomenon could be caused by multiple reasons like radiation-associated hypogonadism and testosterone therapy. Furthermore, factors such as hypermobile testes, horizontal testicular lie, hyperactive cremasteric reflex and defective gubernacular attachment are congenital aspects which can contribute to testicular torsion. Meta-analyses report testicular salvage rates of approximately 90% if de-torsion is completed within 6 hours of the onset of symptoms, 50% at 12 hours and 10 to 25% at 24 hours [17]. For the patient in our case, even with complications, the time spent in the hospital was less than 8 hours with a positive outcome. This signifies the importance of touch and not relying on investigations out of hours for testicular torsion.

## Conclusions

Testicular torsion must always be the foremost differentials to exclude for the cause of acute scrotal pain. By avoiding any delays in diagnosis, the percentage of potential testicular salvage can be greatly increased - especially in the 6-hour window. Based on our case, we have demonstrated that in patients with DMD, it is important to consider that their age for presentation may be over 25 years old, and they may not have a typical pain history due to the condition’s effect on nervous supply to the region. Thus, examination findings have more value in diagnosis. Furthermore, scrotal exploration can be safely and ethically performed via local anaesthetic given the cardio-respiratory sequelae of DMD.
